# Evaluation of Microvascular Changes in the Macula After Uneventful Phacoemulsification Surgery Using Optical Coherence Tomography Angiography

**DOI:** 10.7759/cureus.112147

**Published:** 2026-07-06

**Authors:** Tanie Natung, Hanjabam Dhanashree, Benjamin Nongrum, Jaya Prabha B, Arundhoti Paul

**Affiliations:** 1 Ophthalmology, North Eastern Indira Gandhi Regional Institute of Health and Medical Sciences, Shillong, IND; 2 Optometry, North Eastern Indira Gandhi Regional Institute of Health and Medical Sciences, Shillong, IND

**Keywords:** central sub-field thickness, faz, octa, perfusion density, phacoemulsification, superficial capillary plexus, vessel density

## Abstract

Study design: This was a longitudinal observational study.

Purpose: The purpose of this study was to evaluate whether there are microvascular changes in the retina after uneventful phacoemulsification surgery using optical coherence tomography angiography (OCTA) under topical anesthesia.

Methods: OCTA scans of the 3 mm × 3 mm macula were obtained in 53 eyes to measure the superficial capillary perfusion (SCP) vessel density, perfusion density, foveal avascular zone (FAZ) area, perimeter, circularity, and central subfield thickness (CST) at the pre-operative visit and at one week and one month following phacoemulsification surgery under topical anesthesia in an Indian population.

Results: There was a statistically significant increase in SCP vessel density at one week (17.604 ± 3.3340) and one month (18.725 ± 2.8280) post-operatively compared with the pre-operative visit (16.017 ± 3.6574) (P < 0.001). Similarly, a statistically significant increase was found in SCP perfusion density at one week (33.079 ± 4.8540) and one month (34.585 ± 4.1916) post-operatively compared with the pre-operative visit (30.285 ± 5.6133) (P < 0.001). However, no significant changes in the FAZ or CST parameters were found post-operatively.

Conclusion: The findings suggest that phacoemulsification may influence retinal microcirculation without causing significant structural macular changes. This study adds to the growing body of evidence regarding retinal microvascular alterations after cataract surgery and highlights the utility of OCTA as a non-invasive tool for evaluating post-operative retinal vascular changes.

## Introduction

Cataract is the most common cause of blindness and the second most common cause of moderate to severe vision impairment globally, with data from 2020 highlighting its significant impact on the global population [1]. Phacoemulsification is the gold standard for the treatment of cataract today [2]. Although cataract surgery is performed in the anterior segment of the eye, studies suggest that pulsatile ocular blood flow in the retina may increase following this surgery [3]. The effects of phacoemulsification on the anterior and posterior segments, including corneal edema, uveitis, intraocular pressure changes, pseudophakic macular edema, and retinal detachment, have been widely investigated [4-6]. However, the potential impact of this procedure on the retinal microvasculature remains poorly understood.

Traditional methods of assessing blood flow changes in the retina, such as Doppler ultrasound and fundus fluorescein angiography (FFA), have their own limitations. Doppler ultrasound is mainly employed to examine larger vessels, while FFA is an invasive technique that requires the use of a contrast agent [7]. In contrast, optical coherence tomography angiography (OCTA) is a more recent imaging technique that uses blood flow movement detection instead of dye injection to provide detailed visualization of the retinal and choroidal vascular density [8].

To the best of our knowledge, there are no studies that have specifically evaluated changes in superficial capillary plexus (SCP) vessel density, perfusion density in the macula, foveal avascular zone (FAZ) area, and central subfield thickness (CST) following uneventful phacoemulsification surgery in Indian eyes, despite some studies having been conducted worldwide. Additionally, prior studies have failed to provide conclusive data regarding these changes.

OCTA offers a promising approach to assessing the retinal microvasculature, allowing for the evaluation of various microvascular parameters. Moreover, OCTA allows layer-by-layer visualization of the retinal vasculature and is easily repeatable and reproducible. The ability to study these parameters in detail could provide valuable insights into the previously unexplored phenomenon of microvascular changes following cataract surgery.

This study aims to address this lacuna in knowledge by evaluating microvascular changes in the macula following uneventful phacoemulsification surgery in Indian eyes. The primary objective of this study is to measure SCP vessel density, perfusion density, FAZ area, perimeter and circularity, and central subfield thickness (CST) using OCTA at baseline (pre-operative visit), one week, and one month post-operatively. The findings are likely to aid in a deeper understanding of the effects of cataract surgery on the retinal microcirculation and offer important clinical insights into the post-operative care of patients undergoing this procedure.

## Materials and methods

This longitudinal observational study was conducted in the Department of Ophthalmology of a tertiary care teaching institute in Northeast India after approval from the Institutional Ethics Committee (T132/2023/132). The study adhered to the principles outlined in the Declaration of Helsinki (1964) and was conducted from September 1, 2023, to January 31, 2025. Informed consent was obtained from all patients enrolled in the study.

Based on the mean superficial foveal density (vasculature) before and after phacoemulsification of 13.2% and 15.2%, with standard deviations of 5.2% and 5.0%, respectively [9], with 80% power at a 95% confidence level, the sample size was estimated to be 53 eyes with cataract undergoing phacoemulsification. The calculation was performed using nMaster V2.0 (Christian Medical College, Vellore, India) for a paired t-test. All consecutive eligible patients were included in the study until the sample size was attained.

The inclusion criteria consisted of patients with uncomplicated age-related cataract aged 50 years or older whose intraocular pressure was less than 22 mmHg. The exclusion criteria included patients with any retinopathy that might result in an abnormal microvascular network, such as diabetic retinopathy, hypertensive retinopathy, retinal vascular diseases, and age-related macular degeneration. Patients with glaucoma, an axial length greater than 24.5 mm, poor OCT images (signal strength <6) due to higher-grade cataract, corneal opacity or unstable fixation, previous treatment by laser or photodynamic therapy, any intraocular surgery, any sign of intraoperative or postoperative complications, or unwillingness to participate in the study were also excluded. Patients with systemic hypertension and diabetes without retinopathy were included in the study.

The enrolled patients underwent a comprehensive clinical evaluation for cataract surgery, including a detailed examination of the anterior and posterior segments, followed by OCTA scans. OCTA raster scans centered on the 3 mm × 3 mm macula were obtained using Cirrus HD-OCT 5000 (Carl Zeiss Meditec, Inc., Dublin, California) after pupillary dilation with Tropicamide 0.8% w/v + Phenylephrine 5% w/v solution. The OCT system automatically segments the macula into central, inner, and full rings and measures the SCP vessel density, perfusion density, and FAZ area, perimeter, and circularity. CST was obtained using the same OCT system. Segmentation errors were manually checked and corrected. The central 1 mm diameter circle was defined as the "central ring," the 2 mm annular ring as the "inner ring," and the whole ETDRS 3 mm × 3 mm area as the "full ring." Measurements were taken at baseline (pre-operative visit), one week, and one month following phacoemulsification surgery.

All surgeries were performed by a single expert surgeon (TN) with more than 15 years of experience performing phacoemulsification with PCIOL (posterior chamber intraocular lens) implantation under topical anesthesia (Proparacaine 0.5% w/v eyedrops). A 2.8 mm clear corneal self-sealing incision, capsulorrhexis, hydrodissection, phacoemulsification, and irrigation/aspiration of the residual lens cortex were sequentially performed. A single-piece foldable AcrySof monofocal, trifocal, monofocal toric, or trifocal toric intraocular lens (IOL) (Alcon Laboratories India Private Limited) was implanted in the capsular bag.

Postoperative treatment consisted of eyedrops containing dexamethasone 0.1% w/v and moxifloxacin 0.5% w/v administered six times a day for seven days and tapered every seven days over one month.

Statistical analysis

Statistical analysis was performed using IBM SPSS Statistics for Windows, Version 22 (Released 2013; IBM Corp., Armonk, New York). Categorical variables are presented as frequencies and percentages. Continuous variables were expressed as mean ± standard deviation. The normality of the data was assessed using the Shapiro-Wilk test. As the variables satisfied the assumption of normality, changes in pre-operative and post-operative measurements of SCP vessel density, perfusion density, FAZ parameters, and CST were analyzed using repeated-measures analysis of variance (ANOVA). The assumption of sphericity was evaluated using Mauchly’s test of sphericity, and the Greenhouse-Geisser correction was applied when the assumption of sphericity was violated. Post hoc pairwise comparisons were performed using the Bonferroni correction. A P-value of <0.05 was considered statistically significant.

## Results

The age of the participants ranged from 50 to 80 years, with a mean age of 63.21 ± 8.365 years. The total number of participants was 53, of whom 28 (52.8%) were male and 25 (47.2%) were female. Twenty-seven cases (50.9%) involved the left eye (LE), while 26 cases (49.1%) involved the right eye (RE). A pre-operative best-corrected visual acuity (BCVA) of 6/12 was recorded in 17 eyes (32.1%), 6/18 in 13 eyes (24.5%), 6/36 in nine eyes (17.0%), 6/24 in eight eyes (15.1%), and 6/9 in six eyes (11.3%) (Table 1). The mean signal strength of the OCTA scans was 7.38 ± 1.18 at the pre-operative visit, 9.11 ± 0.92 at the one-week post-operative visit, and 9.42 ± 0.88 at the one-month post-operative visit. 

**Table 1:** Demographic profile of patients in the study population. BCVA: best-corrected visual acuity.

Patient characteristics	Patient data
Age	63.21 ± 8.365 years (50-80 years)
Sex	
Male	28 (52.8%)
Female	25 (47.2%)
Laterality	
Right eye	26 (49.1%)
Left eye	27 (50.9%)
Pre-operative BCVA	
6/12	17 eyes (32.1%)
6/18	13 eyes (24.5%)
6/36	9 eyes (17.0%)
6/24	8 eyes (15.1%)
6/9	6 eyes (11.3%)

Changes in macular vessel density

**Figure 1:**
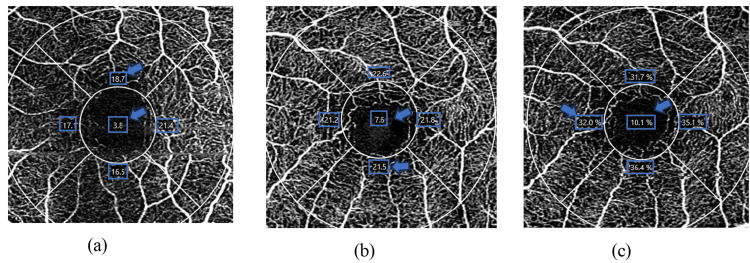
OCTA scans of the 3 mm × 3 mm macula showing vessel density in the SCP in the central and inner rings of the retina at the pre-operative visit (a), one-week post-operative visit (b), and one-month post-operative visit (c) following uneventful phacoemulsification under topical anesthesia. Arrows showing vessel density in SCP in the central and inner rings of the retina. OCTA: optical coherence tomography angiography, SCP: superficial capillary plexus.

The repeated-measures ANOVA demonstrated a statistically significant change in the vessel density of the central, inner, and full 3 mm × 3 mm rings of the macula at one week and one month post-operatively (both P < 0.001) (Table 2). The corresponding OCTA scans are shown in Figure 1. 

The Bonferroni post hoc analysis revealed a statistically significant increase in the macular vessel density of the "central" ring between the pre-operative and one-week post-operative follow-up values (P < 0.001). Similarly, there was a statistically significant increase in the macular vessel density of the central ring between the pre-operative and one-month post-operative follow-up values (P < 0.001). However, there was no statistically significant difference between the one-week and one-month post-operative follow-up values (P = 0.059) (Table 2).

The Bonferroni post hoc analysis revealed a statistically significant increase in the macular vessel density of the "inner" ring between the pre-operative and one-week post-operative follow-up values (P = 0.001), between the pre-operative and one-month post-operative values (P < 0.001), and between the one-week and one-month post-operative values (P = 0.001) (Table 2).

Similarly, the Bonferroni post hoc analysis revealed a statistically significant increase in the vessel density of the "full" 3 mm × 3 mm ring of the macula between the pre-operative and one-week post-operative follow-up values (P = 0.002), between the pre-operative and one-month post-operative follow-up values (P < 0.001), and between the one-week and one-month post-operative follow-up values (P < 0.001) (Table 2).

Changes in macular perfusion density

**Figure 2:**
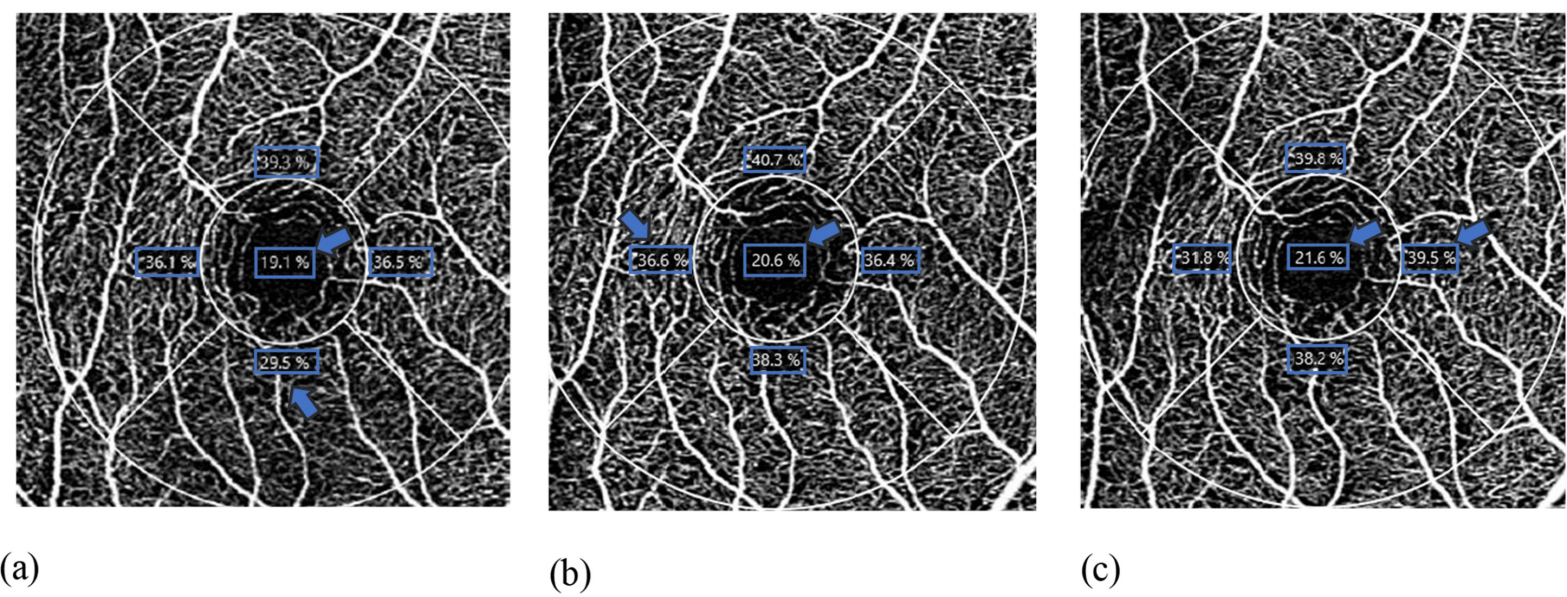
OCTA scans of the 3 mm × 3 mm macula showing perfusion density in the SCP in the central and inner rings of the retina at the pre-operative visit (a), one-week post-operative visit (b), and one-month post-operative visit (c) following uneventful phacoemulsification under topical anesthesia. Arrows show the perfusion density in the SCP (superficial capillary plexus) in the central and inner retina. OCTA: optical coherence tomography angiography, SCP: superficial capillary plexus.

The repeated-measures ANOVA demonstrated a statistically significant change in the perfusion density of the central, inner, and full 3 mm × 3 mm rings of the macula at one week and one month post-operatively (both P < 0.001) (Table 2). The corresponding OCTA scans are shown in Figure 2.

The Bonferroni post hoc analysis revealed a statistically significant increase in the macular perfusion density of the "central" ring between the pre-operative and one-week post-operative follow-up values (P < 0.001). Similarly, there was a statistically significant increase in the macular perfusion density of the central ring between the pre-operative and one-month post-operative follow-up values (P < 0.001). However, there was no statistically significant difference between the one-week and one-month post-operative follow-up values (P = 0.581) (Table 2).

The Bonferroni post hoc analysis revealed a statistically significant increase in the macular perfusion density of the "inner" ring between the pre-operative and one-week post-operative follow-up values (P < 0.001), between the pre-operative and one-month post-operative values (P < 0.001), and between the one-week and one-month post-operative values (P < 0.001) (Table 2).

Similarly, the Bonferroni post hoc analysis revealed a statistically significant increase in the perfusion density of the "full" 3 mm × 3 mm ring of the macula between the pre-operative and one-week post-operative follow-up values (P < 0.001), between the pre-operative and one-month post-operative follow-up values (P < 0.001), and between the one-week and one-month post-operative follow-up values (P = 0.001) (Table 2). 

**Table 2:** Changes in vessel density and perfusion density in the macula, FAZ area, perimeter, and circularity, and CST at the pre-operative visit, one-week post-operative visit, and one-month post-operative visit following uneventful phacoemulsification under topical anesthesia. *Central 1 mm diameter circle in a 3 mm × 3 mm raster scan. ^†^2 mm annular ring in a 3 mm × 3 mm raster scan ^‡ ^The whole ETDRS 3 mm × 3 mm region. FAZ: foveal avascular zone, CST: central subfield thickness, ETDRS: Early Treatment Diabetic Retinopathy Study.

Parameter	Macular region	Pre-operative	Values 1-week post-op	1-month post-op	P-value	Bonferroni post hoc test
Vessel density (mm/mm^2^)	Central ring^*^	7.645 ± 3.0136	8.953 ± 3.1719	9.558 ± 3.2063	<0.001	1 month/1 week > Baseline
	Inner ring^†^	16.979 ± 3.8704	18.775 ± 3.4593	20.070 ± 3.3329	<0.001	1 month>1 week > Baseline
	Full ring^‡^	16.017 ± 3.6574	17.602 ± 3.3340	18.725 ± 2.8280	<0.001	1 month > 1 week > Baseline
Perfusion density (%)	Central ring*	12.419 ± 5.2976	15.047 ± 5.1709	15.464 ± 5.3026	<0.001	1 month/ 1 week > Baseline
	Inner ring^†^	32.068 ± 5.9406	34.906 ± 5.0274	36.747 ± 4.5669	<0.001	1 month >1 week > Baseline
	Full ring^‡^	30.285 ± 5.6133	33.079 ± 4.8540	34.585 ± 4.1916	<0.001	1 month > 1 week > Baseline
FAZ area (mm^2^)		0.3228 ± 0.1025	0.3247 ± 0.09337	0.3294 ± 0.09891	0.759	Not applicable
FAZ perimeter (mm)		2.6515 ± 0.4034	2.6702 ± 0.40842	2.6538 ± 0.53161	0.934	Not applicable
FAZ circularity		0.5770 ± 0.0991	0.5889 ± 0.11825	0.5913 ± 0.11907	0.61	Not applicable
CST (µm)		242.23 ± 18.714	243.17 ± 17.874	244.74 ± 17.684	0.108	Not applicable

Changes in FAZ parameters and CST

The repeated-measures ANOVA showed no statistically significant change in the FAZ area, perimeter, and circularity following uneventful phacoemulsification surgery (P = 0.759, 0.934, and 0.61, respectively) (Table 2).

**Figure 3:**
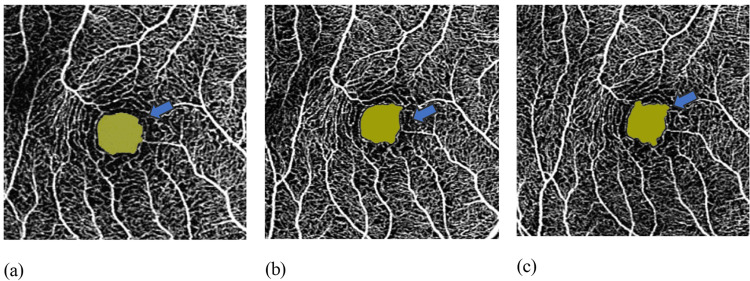
OCTA scans of the 3 mm × 3 mm macula showing the FAZ at the pre-operative visit (a), one-week post-operative visit (b), and one-month post-operative visit (c) following uneventful phacoemulsification under topical anesthesia. Arrows show the FAZ of the retina. OCTA: optical coherence tomography angiography, FAZ: foveal avascular zone.

**Figure 4:**
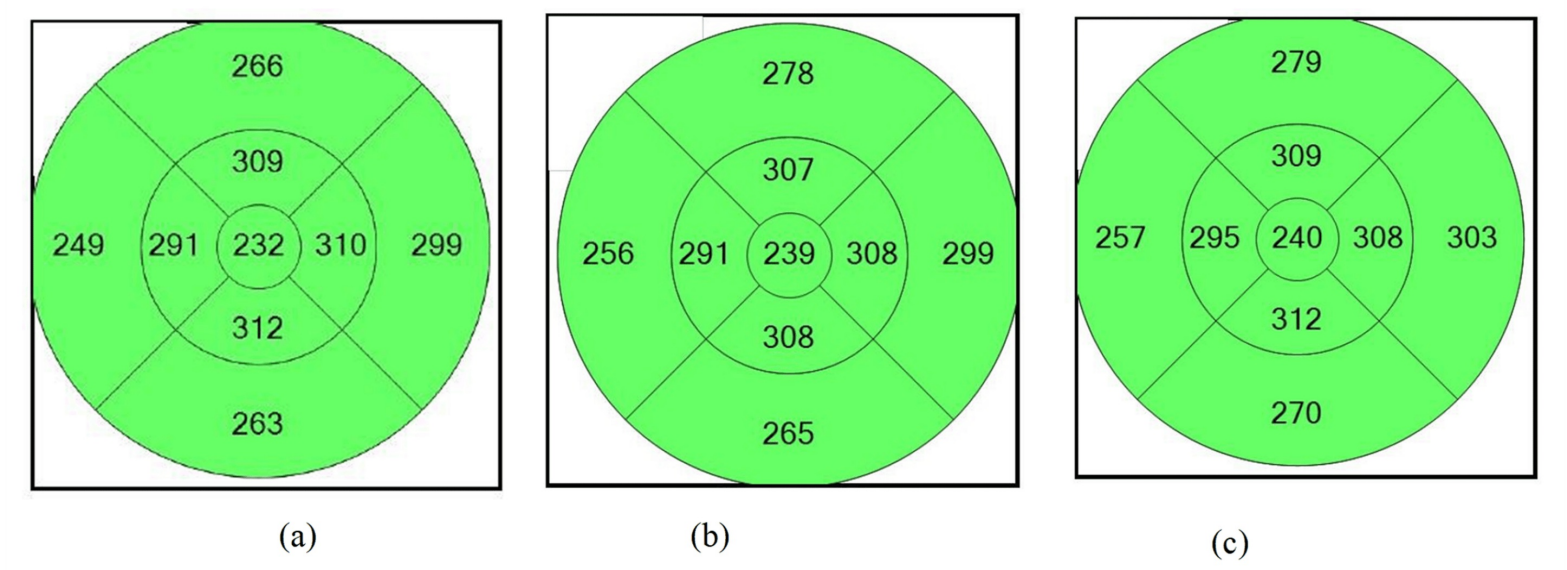
OCT macula scans showing CST changes at the pre-operative visit (a), one-week post-operative visit (b), and one-month post-operative visit (c) following uneventful phacoemulsification under topical anesthesia. OCT: optical coherence tomography, CST: central subfield thickness.

Similarly, there was no statistically significant change in CST over time (P = 0.108) (Table 2). The corresponding OCTA and OCT macula scans are shown in Figures 3, 4. 

## Discussion

This study aimed to evaluate the microvascular changes in the 3 mm × 3 mm macula using parameters such as the vessel density and perfusion density in the SCP, FAZ area, FAZ perimeter, FAZ circularity, and CST after uneventful phacoemulsification surgery under topical anesthesia. The study population comprised 53 participants, of whom 28 (52.8%) were male and 25 (47.2%) were female.

Our study demonstrates a statistically significant increase in both the vessel density and perfusion density of the SCP of the macula following uneventful phacoemulsification surgery, particularly at one week and one month post-operatively. These findings suggest an early and sustained improvement in retinal microcirculation following cataract removal, supporting the hypothesis that cataract surgery positively impacts retinal perfusion.

The progressive increase observed in the central, inner, and full-ring macular vessel density and perfusion density in the SCP aligns with previous studies. Zhao et al. [10] and Baldascino et al. [11] reported similar post-operative enhancements in SCP vessel density, with Zhao attributing these changes to decreased intraocular pressure, post-surgical inflammation, and increased retinal light exposure. Likewise, Özkan et al. [9] and Yu et al. [12] demonstrated marked improvements in SCP and DCP parameters after surgery. Yu et al. stated that these changes could be due to improved image quality of the OCTA scans and a reduction in imaging artifacts [12].

While our study observed no statistically significant change in the foveal avascular zone (FAZ) parameters, there was a slight, non-significant increase in the FAZ area and variability in circularity at the one-week post-operative visit, which returned close to baseline by one month. This transient variability might reflect temporary inflammatory or hemodynamic changes following surgery, as seen in other studies by Özkan et al. and Yu et al. [9,12]. However, studies by Zhao et al. [10], Liu et al. [13], and Nourinia et al. [14] found a statistically significant decrease in the foveal avascular zone at one week, one month, and three months after surgery. Nonetheless, the largely stable FAZ parameters suggest that uneventful phacoemulsification does not cause lasting anatomical disruption to the central macula.

Sodhi et al. conducted a study in the Indian population and found the mean FAZ area, FAZ perimeter, FAZ circularity index, and SCP vessel density to be 0.42 ± 0.23 mm², 3.3 ± 1.0 mm, 0.46 ± 0.1, and 23.87 ± 10.66, respectively. They found that in healthy subjects, a relatively thicker CFT is likely to have better vascularity of the SCP and DCP and a smaller FAZ area [15]. Tan et al. conducted a study on Asian eyes to develop a normative database and found that perfusion density ranges between 30% and 57% in the SCP, 22% and 34% in the deep capillary plexus (DCP), and 36% and 62% in the full retina [16].

Central subfield thickness (CST) showed a mild post-operative increase in our study, though it was not statistically significant. These findings are consistent with previous work by Zhao et al. [10], Liu et al. [13], Georgopoulos et al. [17], Gharbiya et al. [18], and Falcão et al. [19], who reported an early post-operative increase in macular thickness, particularly within the inner retina. These increases have been linked to transient post-surgical inflammation, supported by experimental studies in mice showing upregulation of pro-inflammatory mediators following lens extraction [20]. However, most changes tend to normalize over time, as noted by Pilotto et al. [21]. Natung et al. conducted a study on 400 subjects using spectral-domain OCT to develop a normative database and found that the normal central subfield thickness is 240.40 ± 18.26 µm in healthy Indian eyes [22].

It is also important to consider the optical and physiological factors influencing post-operative imaging and interpretation. The substantial improvement in image quality and signal strength of the scans following cataract removal likely contributes to the observed increase in vascular metrics. As Yu et al. noted, reduction in motion artifacts and improved signal strength post-operatively underscore the influence of pre-operative media opacity on OCTA measurements [12]. However, in our study, statistically significant increases in vessel density and perfusion density parameters were observed not only between the pre-operative and post-operative measurements but also between the one-week and one-month post-operative visits, particularly in the inner and full-ring macular regions. This persistence of statistically significant vascular changes between the two post-operative visits suggests that improved image quality alone may not fully account for the observed findings. Nevertheless, the potential influence of post-operative changes in signal strength and image quality cannot be completely excluded.

Despite the observed improvement in SCP perfusion and vessel density, our findings showed no significant change in the FAZ area or CST. This may suggest a state of improved retinal oxygenation and metabolic support without substantial anatomical remodeling.

Strength and limitations

The strength of our study is that a statistically significant increase in perfusion density and vessel density in the SCP was found using OCTA in the Indian study population, which has not been reported previously. Our study is limited by a relatively small sample size and a short follow-up duration. Longer-term studies with larger populations are required to determine whether the observed microvascular changes persist or normalize over a longer period. Moreover, it would be beneficial to consider parameters such as cumulative phacoemulsification ultrasound energy, ultrasound time, surgical time, and correlation with cataract grade. Additionally, while our focus was on the SCP, changes in the DCP should be explored in future research because of its known susceptibility to metabolic and inflammatory changes. Our study was limited by the unavailability of software to evaluate the DCP.

## Conclusions

The findings suggest that phacoemulsification may influence retinal microcirculation without causing significant structural macular changes. This study adds to the growing body of evidence regarding retinal microvascular alterations after cataract surgery and highlights the utility of OCTA as a noninvasive tool for evaluating post-operative retinal vascular changes.
